# Using real-world data to monitor and improve quality of care in coronary artery disease: results from the Netherlands Heart Registration

**DOI:** 10.1007/s12471-022-01672-0

**Published:** 2022-04-07

**Authors:** Marijke J. C. Timmermans, Saskia Houterman, Edgar D. Daeter, Peter W. Danse, Wilson W. Li, Erik Lipsic, Maaike M. Roefs, Dennis van Veghel, the PCI Registration Committee of the Netherlands Heart Registration and the Cardiothoracic Surgery Registration Committee of the Netherlands Heart Registration

**Affiliations:** 1 Netherlands Heart Registration, Utrecht, The Netherlands; 2 Department of Cardiothoracic Surgery, St Antonius Hospital, Nieuwegein, The Netherlands; 3 Department of Cardiology, Rijnstate Hospital, Arnhem, The Netherlands; 4 Department of Cardiothoracic Surgery, Radboud University Medical Centre, Nijmegen, The Netherlands; 5 Department of Cardiology, University Medical Centre Groningen, Groningen, The Netherlands

**Keywords:** Quality registry, Value-based healthcare, Coronary artery disease, Percutaneous coronary intervention, Coronary artery bypass grafting

## Abstract

Worldwide, quality registries for cardiovascular diseases enable the use of real-world data to monitor and improve the quality of cardiac care. In the Netherlands Heart Registration (NHR), cardiologists and cardiothoracic surgeons register baseline, procedural and outcome data across all invasive cardiac interventional, electrophysiological and surgical procedures. This paper provides insight into the governance and processes as organised by the NHR in collaboration with the hospitals. To clarify the processes, examples are given from the percutaneous coronary intervention and coronary artery bypass grafting registries. Physicians who are mandated by their hospital to instruct the NHR to process their data are united in registration committees. The committees determine standard sets of variables and periodically discuss the completeness and quality of data and patient-relevant outcomes. In the case of significant variation in outcomes, processes of healthcare delivery are discussed and good practices are shared in a non-competitive and safe setting. To create new insights for further improvement in patient-relevant outcomes, quality projects are initiated on, for example, multivessel disease treatment, cardiogenic shock and diagnostic intracoronary procedures. Moreover, possibilities are explored to expand the quality registries through additional relevant indicators, such as resource use before and after the procedure, by enriching NHR data with other existing data resources.

**Supplementary Information:**

The online version of this article (10.1007/s12471-022-01672-0) contains supplementary material, which is available to authorized users.

## Introduction

Cardiovascular diseases are the most important cause of mortality and morbidity in almost all countries of the European Union, with coronary artery disease (CAD) affecting most people [1–3]. Worldwide, quality registries for cardiovascular diseases enable and advance the use of real-world data. Healthcare providers use these real-world data to monitor and benchmark the quality of care, to implement improvement initiatives and to conduct scientific research [4–6].

In the Netherlands, structural registration and monitoring of healthcare outcomes is performed on a compulsory basis, enforced by law or the government [7, 8]. Also, the Dutch Society of Cardiology (NVVC) and the Dutch Society of Cardiothoracic Surgeons (NVT) can define mandatory elements in the registry as part of their quality policy. In addition to these external incentives to register, the concept of value-based healthcare (VBHC) is of increasing interest to healthcare providers in their efforts to optimise the quality of care [9]. Key to measuring value is defining condition-specific outcomes that matter to patients [10]. Public reporting is believed to contribute to quality improvement [11, 12].

To effectively facilitate the ambition of care providers to structurally measure and improve outcomes, in 2017 in collaboration with the NVVC and the NVT three separate quality registries (*Begeleidingscommissie Hartinterventies Nederland* [Supervisory Committee for Cardiac Interventions in the Netherlands], the National Cardiovascular Data Registry [NCDR] and Meetbaar Beter) merged into one national registry: the Netherlands Heart Registration (NHR). Within the NHR, cardiologists and cardiothoracic surgeons register baseline, procedural and outcome data across all invasive cardiac interventional, electrophysiological and surgical procedures. Cardiac anaesthetists participate and register data as part of the cardiac surgery registry. The NHR covers over 1.5 million cardiac procedures across the Netherlands, and over 80,000 procedures are added yearly. Through public reporting, the NHR serves cardiac patients, healthcare providers and policy makers in making outcome data transparent [13].

The aim of this paper is to provide insight into the governance and processes as organised by the NHR in collaboration with the hospitals. Coronary artery bypass grafting (CABG) and percutaneous coronary intervention (PCI), the two treatments with the highest incidence among patients with cardiovascular diseases, are used as sample models for further clarification.

## The NHR

The NHR is a non-profit organisation which aims to contribute to quality improvement and safety in cardiac care by facilitating quality registries. For this purpose, the NHR processes personal data of patients by order of the hospitals. The legal basis is covered by the Healthcare Quality, Complaints and Disputes Act (Dutch: Wkkgz), which obligates healthcare providers to evaluate and improve their own quality [7, 14]. Participating hospitals are responsible for data collection and registration and remain the owner of the data they submit. The NHR analyses patient data, provides online dashboards and reports relevant outcome indicators in yearly, publicly accessible reports [13].

### Registration committees

Physicians who are mandated by their hospital to instruct the NHR to process their data are united in registration committees. To date, seven registration committees are operational: Cardiothoracic surgery, PCI, Transcatheter heart valve interventions, Ablation of atrial fibrillation, Pacemaker/ICD, Heart failure and Atrial fibrillation. In these registration committees standard sets of patient-relevant outcome measures, process variables and patient characteristics are constructed, using a fixed, step-wise approach [15, 16]. The committees collaborate closely in order to align the standard sets of variables. Completeness and quality of data and outcomes are discussed. In the case of clinically relevant and/or statistically significant variation of outcomes, processes of healthcare delivery are discussed and good practices are shared. In addition, quality improvement projects and scientific research are initiated and conducted.

The collaboration of cardiologists, cardiothoracic surgeons and cardiac anaesthetists within registration committees, who play a leading role in defining the standard sets of variables and initiate relevant quality projects, is a unique feature of the NHR in comparison to international registries for cardiology and cardiothoracic surgery [17–20]. Many other processes, like the data quality assurance systems and methods for data analyses, are largely comparable.

### Registration, innovation and scientific research

The NHR distinguishes three primary processes: registration, innovation and scientific research. Regarding registration, the NHR facilitates for all hospitals in the Netherlands the process of registering the standard sets of variables that are mandatory by law or required by the quality policies of the NVVC and NVT. An example of obligations by law is the standard set for PCI, which is part of the Transparency Calendar (*Transparantiekalender*), a mandatory register initiated in 2014 by the Dutch government with the aim of creating insights into the quality of care for a selection of diseases [8]. Regarding the quality policy, the NVT developed mandatory funnel plots in which risk-adjusted mortality rates per centre are publicly reported on an annual basis for the most common cardiac surgical procedures, including CABG. In a secure online environment, hospitals have the possibility to monitor their own data in relation to aggregated data from the other Dutch hospitals.

Within the process of innovation, new registries are initiated (e.g. heart failure and atrial fibrillation), collaboration is established with other data sources (e.g. Dutch Hospital Data, Vektis) to reduce the registration burden, and a VBHC programme is facilitated. Within the VBHC programme, patient-relevant outcomes corrected for patient characteristics are presented yearly in a publicly accessible report [12]. Participation in the programme is voluntary. Currently, 26 out of 30 hospitals (including 15 of the 16 centres with onsite cardiothoracic surgery) take part in this programme.

All participating hospitals can request use of data available within the NHR to perform observational scientific research. The registration committees and a scientific council, consisting of a representative group of physicians from the participating hospitals, advise the board of the NHR regarding the medical relevance, scientific methods and feasibility of the studies. If a request is granted, data can be used in aggregated form. Non-aggregated data can only be used with the permission of the hospitals concerned and/or the individual patients. In the near future, an infrastructure for prospective research (both observational and experimental, i.e. registry-based randomised clinical trials [RB-RCTs]) will become available.

### Data quality assurance system

The value of registries like the NHR strongly depends on the quality of the data [21]. Under-reporting of events can, for example, lead to wrong decision making. The NHR aims to minimise those risks by having a data quality assurance system in place, as a part of the NEN-7510 certificate the NHR holds, which provides frameworks for information security for healthcare organisations and associated organisations, such as providers of software and IT services [22]. To ensure validity and consistency of the data, the NHR provides a detailed data dictionary, which contains definitions and coding guidelines for all variables [23]. Data extracted from the electronic medical records are submitted to the NHR in a secure online environment and transferred in an encrypted format to a central server. Data are validated using different methods, giving the hospitals the opportunity to verify the uploaded data before their use for public reporting or scientific research. The hospitals receive an automated quality report from the system containing information about errors or inconsistencies in the data. In addition, the hospitals have the possibility to monitor their own data in the secure online environment and to compare them to aggregated data from the other Dutch hospitals. Subsequently, the data analysts of the NHR conduct supplemental quality checks and design quality reports. In addition to these methods to validate the data, each year a monitoring visit is conducted by an independent auditor. During the visit, a selection of data submitted to the NHR is compared with the information in the medical records. Results of the yearly data monitoring visits and discussions within the registration committees indicate that the quality of the PCI and CABG registration is very high, with an accuracy of above 95% for almost all variables [24].

### Standard sets of variables

Participating hospitals register standard sets of variables, for example for PCI and CABG, which are constructed by the corresponding registration committees following a fixed approach [15, 16]. In short, outcome measures are selected based on the following criteria: (1) patient relevance, defined as the impact on patient mortality and/or morbidity and/or quality of life; (2) incidence of the outcome; and (3) the level of impact health professionals can have on the outcome. In addition, the feasibility of data collection and the quality of the outcome definition are taken into account. Tab. 1 and 2 show a selection of the standard sets of outcome measures, process variables and patient characteristics for PCI and CABG. The complete sets of variables are described in detail in the data dictionary [23].

**Table 1:** Uncorrected trends in outcome measures and patient characteristics for percutaneous coronary intervention (*PCI*), 2015–2020

	National mean (SD) or proportion per year					
	2015 (*n* = 40,170)	2016 (*n* = 41,029)	2017 (*n* = 40,296)	2018 (*n* = 40,132)	2019 (*n* = 40,715)	2020^a^ (*n* = 37,732)
*Outcome measures*						
30-day mortality						
– All PCI	2.7%	2.7%	2.7%	2.7%	2.6%	2.9%
– Elective	0.6%	0.7%	0.5%	0.8%	0.6%	0.8%
– Non-STEMI	1.8%	1.9%	1.8%	1.8%	1.9%	1.8%
– STEMI	6.0%	6.0%	6.2%	6.1%	5.8%	6.2%
1-year mortality						
– All PCI	5.7%	5.5%	5.6%	5.7%	5.5%	NA
– Elective	3.1%	3.4%	3.3%	3.9%	3.5%	NA
– Non-STEMI	5.5%	5.2%	5.2%	5.3%	5.4%	NA
– STEMI	8.7%	8.2%	8.7%	8.6%	8.3%	NA
Long-term survival (≤5 years)	Presented in survival curves					
Acute CABG (≤24 h)	0.2%	0.3%	0.3%	0.1%	0.2%	0.2%
Myocardial infarction (≤30 days)	0.9%	0.7%	0.6%	0.6%	0.8%	0.8%
Target vessel revascularisation (≤1 year)	7.0%	6.2%	6.1%	6.0%	6.4%	NA
Quality of life^b^						NA
– Physical health (% improved <1 year)	54.5%	60.1%	65.0%	61.3%	52.2%	NA
– Mental health (% improved <1 year)	57.5%	51.4%	54.2%	55.8%	50.4%	NA
*Procedural variables*						
Access route						
– Transradial	–	–	–	83.7%	86.8%	88.2%
– Transfemoral	–	–	–	16.1%	12.9%	11.5%
Number of treated vessels (% single-vessel PCI)						
– Elective	–	–	–	58.0%	61.4%	64.5%
– Non-STEMI	–	–	–	62.1%	64.0%	65.9%
– STEMI	–	–	–	85.2%	86.9%	87.1%
*Patient characteristics*						
Age (years), mean (SD*)*	66 (12)	66 (12)	66 (12)	67 (12)	67 (11)	67 (11)
Cardiogenic shock	2.8%	2.5%	2.3%	2.7%	2.9%	3.3%
Chronic total occlusion	5.5%	5.4%	6.1%	5.8%	5.2%	4.8%
Diabetes mellitus	21.1%	21.2%	21.8%	21.4%	22.4%	21.5%
Gender (male)	72.3%	71.6%	71.9%	72.2%	72.5%	72.6%
Indication PCI						
– Elective	34.2%	35.0%	36.1%	37.6%	36.4%	32.1%
– Non-STEMI	33.8%	32.9%	33.8%	33.2%	34.2%	36.1%
– STEMI	31.9%	32.1%	30.2%	29.2%	29.4%	31.8%
Left ventricular ejection fraction (if available)						
– > 50%	59.3%	63.4%	62.1%	61.4%	68.6%	64.9%
– 30–50%	33.3%	29.8%	32.4%	33.9%	25.7%	28.5%
– < 30%	7.4%	6.8%	5.5%	4.8%	5.7%	6.7%
Multivessel disease	48.3%	46.9%	47.7%	46.4%	50.9%	52.6%
Out-of-hospital cardiac arrest	3.9%	3.7%	3.4%	3.6%	3.6%	3.7%
Previous CABG	10.4%	9.7%	9.9%	9.0%	9.0%	9.0%
Previous myocardial infarction	23.0%	21.3%	22.4%	20.2%	21.1%	21.2%
Renal insufficiency (eGFR <60)	21.7%	22.8%	23.5%	23.6%	23.8%	23.2%

*STEMI* ST-segment elevation myocardial infarction, *CABG* coronary artery bypass grafting, *eGFR* estimated glomerular filtration rate, *NA* not applicable because, when the data were uploaded (May 2021), the cohort of patients treated in the year in question had not completed follow-up

^a^Numbers may be distorted because of the impact of the COVID-19 pandemic

^b^Measured by the SF-36 or SF-12 questionnaire, at baseline and between 10 and 14 months after treatment

**Table 2:** Uncorrected trends in outcome measures and patient characteristics for isolated coronary artery bypass grafting (*CABG*), 2013–2020

	National mean (SD) or proportion per year							
	2013 (= 7929)	2014 (*n* = 7754)	2015 (*n* = 7538)	2016 (*n* = 7289)	2017 (*n* = 7255)	2018 (*n* = 6610)	2019 (*n* = 7379)	2020^a^ (*n* = 6542)
*Outcome measures*								
30-day mortality	1.2%	1.3%	1.3%	1.4%	1.3%	1.4%	1.0%	1.3%
120-day mortality	1.7%	1.7%	1.9%	2.0%	1.7%	2.1%	1.4%	1.7%
1‑year mortality	2.6%	2.5%	2.7%	2.8%	2.6%	2.9%	2.5%	NA
Long-term survival (≤5 years)	Presented in survival curves							
Surgical re-exploration (≤30 days)	4.8%	3.8%	3.5%	3.8%	4.0%	4.5%	4.6%	4.4%
Cerebrovascular accident with residual deficit during hospital stay	0.5%	0.7%	0.7%	0.7%	0.8%	0.7%	0.7%	0.6%
Deep sternal wound infection (≤30 days)	0.9%	0.9%	0.8%	1.1%	0.9%	1.1%	1.1%	0.9%
Coronary re-intervention (≤5 years)	Presented in survival curves							
Quality of life^b^								
– Physical health (% improved <1 year)	57.8%	60.0%	65.6%	61.8%	61.0%	63.6%	58.5%	NA
– Mental health (% improved <1 year)	47.1%	48.2%	56.8%	50.2%	44.4%	54.0%	51.2%	NA
*Procedural variables*								
Length of hospital stay^c^: days, median (IQR)	5 (4–7)	5 (4–6)	5 (4–6)	5 (4–6)	5 (4–7)	5 (4–6)	5 (4–6)	5 (4–6)
Waiting time^d^: days, median (IQR)	13 (6–32)	21 (7–42)	20 (7–47)	17 (7–39)	21 (7–44)	22 (7–45)	30 (10–54)	34 (14–64)
Off-pump	14.7%	16.1%	16.5%	18.4%	19.5%	14.6%	15.6%	16.8%
*Patient characteristics*								
Age (years), mean (SD)	66 (10)	66 (10)	67 (9)	66 (10)	67 (9)	67 (9)	67 (9)	67 (9)
Chronic lung disease	9.9%	10.4%	10.6%	9.8%	9.0%	8.6%	9.1%	8.1%
Diabetes mellitus	25.6%	24.9%	26.0%	26.8%	25.7%	25.2%	28.6%	28.2%
Sex (male)	79.3%	79.6%	80.6%	79.9%	81.3%	81.6%	81.3%	81.6%
Left ventricular ejection fraction								
– > 50%	73.7%	72.1%	71.4%	72.8%	72.6%	72.0%	70.7%	67.6%
– 30–50%	21.9%	24.0%	24.8%	23.1%	24.0%	24.8%	25.9%	28.5%
– < 30%	3.8%	3.0%	3.1%	3.6%	3.4%	3.2%	3.4%	3.9%
Logistic EuroSCORE I (high >19.5%)	4.1%	3.6%	3.2%	2.9%	2.9%	2.7%	2.5%	2.9%
Logistic EuroSCORE II (high >9.5%)	–	–	2.7%	2.5%	2.2%	2.5%	2.0%	2.3%
Multivessel disease	88.2%	89.0%	92.0%	92.9%	91.9%	86.5%	89.7%	88.9%
Previous cardiac surgery	2.7%	1.9%	1.7%	1.8%	1.4%	1.5%	1.1%	1.4%
Renal insufficiency (eGFR <60)	20.1%	20.9%	20.1%	22.2%	22.1%	22.6%	21.6%	19.7%
Urgency of the procedure (emergency + salvage)	6.9%	6.5%	6.0%	6.5%	6.1%	5.4%	5.1%	6.1%

*NA* not applicable because, when the data were uploaded (March 2021), the cohort of patients who were treated in the year in question had not completed follow-up, *SD* standard deviation, *IQR* interquartile range, *eGFR* estimated glomerular filtration rate

^a^Numbers may be distorted because of the impact of the COVID-19 pandemic

^b^Measured by the SF-36 or SF-12questionnaire, at baseline and between 10 and 14 months after treatment

^c^Calculated as the number of days between the date of the CABG and the date of discharge from the centre which performed the CABG

^d^Calculated as the number of days between the date of acceptance by the heart team and the date of the CABG for elective patients only

## Results and trends in the treatment of CAD by CABG or PCI

Since the start of the NHR in 2017, over 1.5 million cardiac procedures have been registered, of which about 700,000 are PCIs (1995–2021, with nationwide coverage from 2015) and about 200,000 isolated CABGs (1995–2021, with nationwide coverage from 2007). Completeness of mandatory variables was 98% for PCI and 99% for isolated CABG in 2020.

**Fig. 1:**
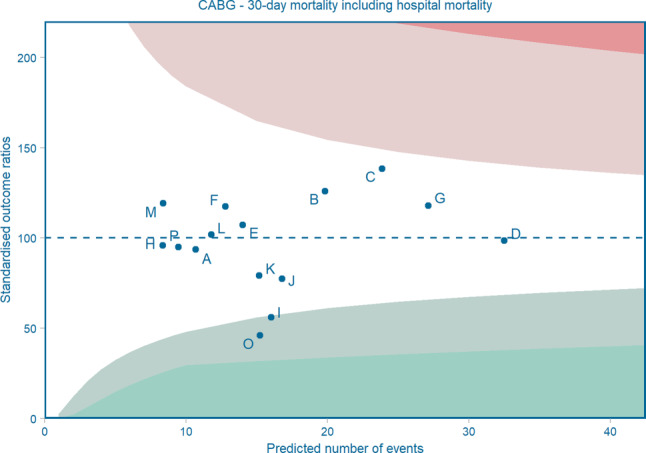
Mandatory Dutch Society of Cardiothoracic Surgeons funnel plot for 30-day mortality after isolated coronary artery bypass grafting (*CABG*) during a 3-year period (2018–2020). C‑statistic = 0.81 (good); years included = 2018–2020. Risk-adjusted for: EuroSCORE II. *A* Amsterdam University Medical Centre, University of Amsterdam, Amsterdam; *B* Amphia, Breda; *C* St. Antonius Hospital, Nieuwegein; *D* Catharina Hospital, Eindhoven; *E* Erasmus Medical Centre, Rotterdam; *F* Haga Hospital, Den Haag; *G* Isala, Zwolle; *H* Leiden University Medical Centre, Leiden; *I* Medical Centre Leeuwarden, Leeuwarden; *J* Medical Spectrum Twente, Enschede; *K* Maastricht University Medical Centre, Maastricht; *L* OLVG, Amsterdam; *M* Radboud University Medical Centre, Nijmegen; *N* University Medical Centre Groningen, Groningen; *O* University Medical Centre Utrecht, Utrecht; *P* Amsterdam University Medical Centre, VU Medical Centre, Amsterdam. (*Note*: The figure is an example of analyses as performed by the NHR and discussed within the cardiothoracic surgery registration committee. To obtain a more complete overview of the clinical outcomes of the hospitals regarding isolated CABG, the annual report of the NHR can be accessed)

**Fig. 2:**
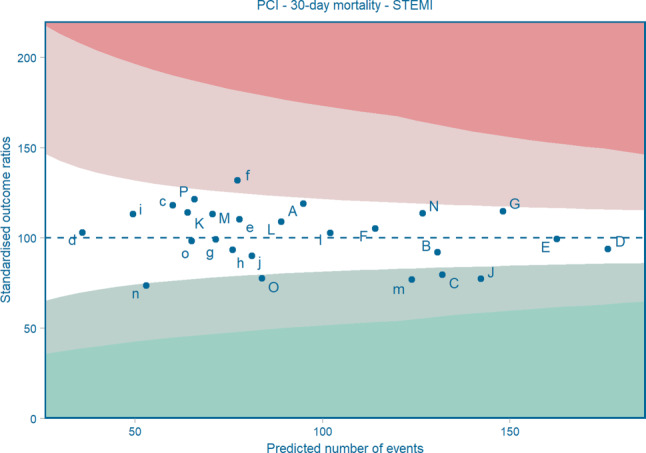
Funnel plot for 30-day mortality after percutaneous coronary intervention (*PCI*) in patients with ST-segment elevation myocardial infarction during a 5-year period (2016–2020). C‑statistic = 0.88 (good); years included = 2016–Q3 2020. Risk-adjusted for: age, cardiogenic shock, chronic total occlusion, diabetes, multivessel disease, out of hospital cardiac arrest, previous CABG, previous myocardial infarction, renal insufficiency, sex and year of the intervention. *A* Amsterdam University Medical Centre, University of Amsterdam, Amsterdam; *B* Amphia, Breda; *C* St. Antonius Hospital, Nieuwegein; *D* Catharina Hospital, Eindhoven; *E* Erasmus Medical Centre, Rotterdam; *F* Haga Hospital, Den Haag; *G* Isala, Zwolle; *I* Medical Centre Leeuwarden, Leeuwarden; *J* Medical Spectrum Twente, Enschede; *K* Maastricht University Medical Centre, Maastricht; *L* OLVG, Amsterdam; *M* Radboud University Medical Centre, Nijmegen; *N* University Medical Centre Groningen, Groningen; *O* University Medical Centre Utrecht, Utrecht; *P* Amsterdam University Medical Centre, VU Medical Centre, Amsterdam; *c* Elisabeth-TweeSteden Hospital, Tilburg; *d* Haaglanden Medical Centre, Den Haag; *e* Jeroen Bosch Hospital, ’s-Hertogenbosch; *f* Maasstad Hospital, Rotterdam; *g* Meander Medical Centre, Amersfoort; *h* Noordwest Hospital Group, Alkmaar; *i* Rijnstate, Arnhem; *j* Tergooi, Blaricum; *m* VieCuri Medical Centre, Venlo; *n* ZorgSaam Hospital, Terneuzen; *o* Zuyderland Medical Centre, Heerlen. (*Note:* The figure is an example of analyses as performed by the NHR and discussed within the PCI registration committee. To obtain a more complete overview of the clinical outcomes of the hospitals regarding PCI, the annual report of the NHR can be accessed)

To observe relevant trends in patient characteristics and clinical outcomes and to be able to compare centre-specific performances, both uncorrected analyses and analyses with correction for the most relevant patient characteristics are performed. Uncorrected trends in outcome measures and patient characteristics for the past years are presented in Tab. 1 (PCI) and Tab. 2 (CABG). Analyses corrected for patient characteristics are particularly depicted in funnel plots. For each centre the number of expected cases (calculated by means of multivariable regression analysis) is plotted against the percentage of the standardised number of cases (calculated by dividing the number of observed cases by the number of expected cases multiplied by 100). A mean ratio for all centres together is calculated with corresponding 95 and 99% confidence intervals, to be able to assess which centres deviate significantly from the national mean. A C-statistic is presented to indicate the discriminatory power of the predictive model [25]. For outcomes with long-term follow-up, like long-term survival, coronary re-intervention and recurrence of myocardial infarction within 5 years, results are presented in risk-adjusted Kaplan Meier curves. For that purpose, multivariable Cox proportional hazard analysis is performed, with risk adjustment for the selected patient characteristics. All figures are updated yearly and discussed within the registration committees. Figs. 1 and 2 show a selection of results to indicate how the standard set is presented and published to optimally facilitate quality control and benchmarking within the centres. All other tables and figures are available in the online publicly accessible report [13].

**Fig. 3:**
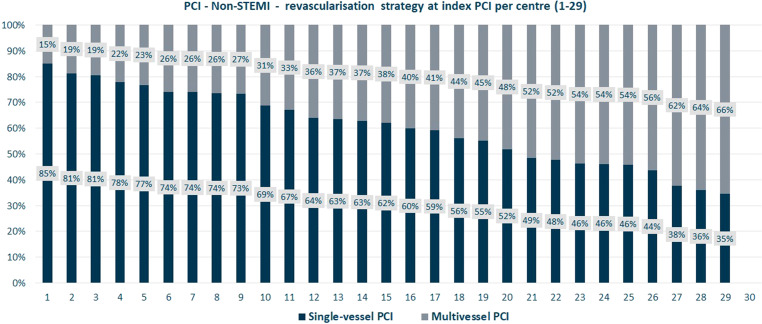
The number of vessels treated during the index percutaneous coronary intervention in non-ST-segment elevation myocardial infarction patients with multivessel disease, per centre (*1–29*)

The NHR conducts additional analyses at the request of the centres to further explore the results. For example, analyses are performed on the variation between centres regarding the choice of access for primary PCI (transradial vs transfemoral access) and the relation of that choice with outcomes. While transradial access is considered the gold standard [26–28], the choice of this access route varied from 59 to 97% among centres. Also, analyses have been performed to create insight into the treatment strategy in patients with multivessel disease (MVD) and the variation between centres, as the optimal revascularisation strategy is debatable (multivessel PCI versus culprit-only PCI), especially for non-ST-segment elevation myocardial infarction (non-STEMI) patients [29, 30]. Data analyses showed that in STEMI patients, about 16% of all index PCIs concerned multivessel PCI (variation between centres 5–53%). In non-STEMI, this accounts for about 40% (variation between centres 15–66%) (Fig. 3). To further explore this variation, a questionnaire has been developed in order to gain more insight into the treatment strategies of the individual centres.

## Future perspectives

Constant monitoring and further improvement of patient-relevant outcomes are of increasing importance to healthcare providers. Besides, public reporting of outcomes is becoming increasingly common among the healthcare systems worldwide, as it has been proposed as a mechanism to provide more transparency and accountability of healthcare providers, and thereby enhances trust between patients, regulators, health insurance companies and care providers [31, 32]. Currently, almost all eligible hospitals (26 out of 30, including 15 of the 16 centres with onsite cardiothoracic surgery) voluntarily participate in the VBHC programme, which indicates that within the Netherlands, heart care is at the forefront of VBHC implementation. Physicians have the possibility to monitor their own data in a secure online environment and to compare them to aggregated data from other hospitals. In addition, benchmark analyses performed by the NHR, such as the funnel plots, form an important base for the evaluation of clinical outcomes in relation to the outcomes of other centres.

Both for PCI and CABG, the incidence of the structurally measured outcomes in Dutch hospitals is relatively low and stable over time, and only little variance is observed between centres. However, measuring those outcomes is still considered relevant from the perspective of quality and safety monitoring. In addition, observing trends over time can also lead to relevant new insights. For example, the cardiothoracic surgery registration committee recently initiated additional research on the slight decrease in the percentage of women who are treated with an isolated CABG.

In 2021, the registration committees started several initiatives in order to create new relevant insights to further improve the quality of cardiac care. For example, an additional registry was initiated for patients with cardiogenic shock following PCI. With an incidence of shock of about 4% within the PCI population and mortality rates of around 50%, it is considered essential to gain more insight into patient characteristics, process variables, treatment outcomes and practice variation [33, 34]. Another example which can provide a new impulse for further improvement of patient-relevant outcomes is the introduction of a registry for isolated and PCI-combined diagnostic intracoronary procedures. Besides, additional analyses have been proposed in order to generate more in-depth insight into subgroups of patients. An example is the analyses on the variation of the revascularisation strategy in patients with MVD, as described above. Also, analyses on inter-physician variability in addition to the common benchmark analyses on a centre level are of increasing interest.

In addition to these in-depth analyses and additional registries, the registration committees and the NHR search for possibilities to expand the quality registries through additional relevant indicators, while limiting the registration burden. By enriching data with other existing data resources like the national health insurance database (Vektis) and the national registry for hospital care (Dutch Hospital Data) [35], the registration committees might be able to create new insights into, for example, resource use before and after the procedure, length of hospital stay and re-admission rates. Also, linking NHR data to other national quality registries, like the Dutch National Intensive Care Evaluation (NICE) Registry, can provide valuable insights without an additional administrative burden [36].

Risk-averse behaviour is considered a potential unintended consequence of public reporting [37, 38]. For example, a study found that after introducing public reporting in Massachusetts and New York, patients who presented with STEMI, cardiac arrest or cardiogenic shock were less likely to undergo PCI than in non-reporting states [39, 40]. Although there are currently no signals of risk-averse behaviour within Dutch heart care, it is essential that any adverse response to public reporting is mitigated. An important step might be a transition from an intervention-oriented registry to a disease-oriented registry, also including, for example, patients with CAD that are not being treated with PCI or CABG. Previous projects showed that this transition is complex, as a significant proportion of the patients with CAD who are treated conservatively are not referred to heart teams and cannot easily be included in the NHR. Recently, new steps have been taken by the introduction of a registry for patients with acute coronary syndrome. Another strategy to mitigate the risk of risk-averse behaviour is to earmark and possibly separately analyse extremely high-risk patients. This strategy will be further explored by the cardiothoracic surgery registration committee.

Structural data registration in electronic patient records is developing quickly. This is considered an essential step to effectively facilitate quality registries like the NHR and to reduce the administrative burden. Besides, this development provides new opportunities to conduct relevant (scientific) evaluation based on real-world data in an efficient manner. Some examples of patient-relevant evaluations, such as the treatment strategy for MVD, have been described above. In addition, the concept of RB-RCTs has received increasing attention in the medical literature, as it has the potential to combine the advantages of randomisation (high internal validity) with the advantages of registries (low expense, high external validity) [41, 42]. Patients can be randomly allocated, with most of the relevant baseline medical history already recorded, minimising the need for additional data collection and onsite monitoring. Currently, the NHR is developing an infrastructure for prospective research that is built up on top of the registry, such as the RB-RCTs.

In conclusion, data for PCI and isolated CABG as registered within the NHR are almost complete. Results of the yearly data monitoring visits and discussions within the registration committees indicate that the data quality is relatively high, with an accuracy of above 95% for almost all variables for PCI. The registration committees have proven to be a valuable platform to discuss data quality, outcomes and processes of healthcare delivery in a non-competitive and safe setting. Using real-world data on a national and hospital level to measure and improve patient-relevant outcomes has proved to be feasible and valuable. Additional data analyses, new quality projects and enrichment of data with other existing data resources may create new relevant insights to further improve the quality of cardiac care in the Netherlands.

## Supplementary Information

A complete list of physicians members of the Cardiothoracic Surgery Registration Committee of the NHR as well as a complete list of physicians members of the PCI Registration Committee of the NHR will be found as Supplementary Electronic Material (ESM).
