# Supplementary material for: Using real-world data to monitor and improve quality of care in coronary artery disease: results from the Netherlands Heart Registration
Source: Neth Heart J. 2022 Apr 7;30(12):546–56. doi: 10.1007/s12471-022-01672-0 (PMC8988537; doi:10.1007/s12471-022-01672-0)
Supplement: Supplementary file 1 — A complete list of physicians members of the Cardiothoracic Surgery Registration Committee of the NHR as well as a complete list of physicians members of the PCI Registration Committee of the NHR will be found as Supplementary Electronic Material (ESM). [file 12471_2022_1672_MOESM1_ESM.docx]

***The following physicians are members of the Cardiothoracic Surgery Registration Committee of the NHR***

They represent the hospitals that have provided the CABG data for this study: S. Bramer, Amphia Hospital, Breda; W.J.P. Van Boven, Amsterdam University Medical Centre, University of Amsterdam, Amsterdam; A.B.A. Vonk, Amsterdam University Medical Centre, VU Medical Centre, Amsterdam; B.M.J.A. Koene, Catharina Hospital, Eindhoven; J.A. Bekkers, Erasmus Medical Centre, Rotterdam; G.J.F. Hoohenkerk, Haga Hospital, Den Haag; A.L.P. Markou, Isala, Zwolle; A. de Weger, Leiden University Medical Centre, Leiden; P. Segers, Maastricht University Medical Centre, Maastricht; F. Porta, Medical Centre Leeuwarden, Leeuwarden; R.G.H. Speekenbrink, Medical Spectrum Twente, Enschede; W. Stooker, OLVG, Amsterdam; W.W.L. Li, Radboud University Medical Centre, Nijmegen; E.J. Daeter, St. Antonius Hospital, Nieuwegein; N.P. van der Kaaij, University Medical Centre Utrecht, Utrecht; Y.L. Douglas; University Medical Centre Groningen, Groningen.

***The following physicians are members of the PCI Registration Committee of the NHR***

They represent the hospitals that have provided the PCI data for this study: M. Scholte, Albert Schweitzer Hospital, Dordrecht; M. Meuwissen, Amphia, Breda; J.P. Henriques, Amsterdam University Medical Centre, University of Amsterdam, Amsterdam; K.M.J. Marques, Amsterdam University Medical Centre, VU Medical Centre, Amsterdam; K. Teeuwen, Catharina Hospital, Eindhoven; H. Al Hashimi, Canisius Wilhelmina Hospital, Nijmegen; M. Magro, Elisabeth TweeSteden Hospital, Tilburg; J. Daemen, Erasmus Medical Centre, Rotterdam; B.J. Sorgdrager, Haaglanden

Medical Centre, Den Haag; C.E. Schotborgh, Haga Hospital, Den Haag; V. Roolvink, Isala, Zwolle;

J. Polad, Jeroen Bosch Hospital, ’s-Hertogenbosch; R. Scherptong, Leiden University Medical Centre, Leiden; M. van der Ent, Maasstad Hospital, Rotterdam; A.J.W. van ’t Hof, Maastricht University Medical Centre, Maastricht; F. Spano, Meander Medical Centre, Amersfoort; J. Brouwer, Medical Centre Leeuwarden, Leeuwarden; M.G. Stoel, Medical Spectrum Twente, Enschede; A. Dedic, Noordwest Hospital Group, Alkmaar; G. Amoroso, OLVG, Amsterdam; C. Camaro, Radboud University Medical Centre, Nijmegen; P.W. Danse, Rijnstate, Arnhem; J.P. van Kuijk, St. Antonius Hospital, Nieuwegein; E.K. Arkenbout, Tergooi, Blaricum; W.T. Ruifrok, Treant Zorggroep, Scheper Hospital, Emmen; A. Kraaijeveld, University Medical Centre Utrecht, Utrecht; E. Lipsic, University

Medical Centre Groningen, Groningen; S. Aydin, VieCuri Medical Centre, Venlo; R. Erdem, ZorgSaam

Hospital, Terneuzen; A.J.W. van ’t Hof, Zuyderland Medical Centre, Heerlen.
